# Lymphocyte Counts and Multiple Sclerosis Therapeutics: Between Mechanisms of Action and Treatment-Limiting Side Effects

**DOI:** 10.3390/cells10113177

**Published:** 2021-11-15

**Authors:** Stefanie Fischer, Undine Proschmann, Katja Akgün, Tjalf Ziemssen

**Affiliations:** Center of Clinical Neuroscience, Department of Neurology, University Clinic Carl-Gustav Carus, Dresden University of Technology, Fetscherstr. 74, 01307 Dresden, Germany; Stefanie.Fischer@uniklinikum-dresden.de (S.F.); Undine.Proschmann@uniklinikum-dresden.de (U.P.); Katja.Akguen@uniklinikum-dresden.de (K.A.)

**Keywords:** multiple sclerosis, lymphocyte counts, mechanism of action, adverse event

## Abstract

Although the detailed pathogenesis of multiple sclerosis (MS) is not completely understood, a broad range of disease-modifying therapies (DMTs) are available. A common side effect of nearly every MS therapeutic agent is lymphopenia, which can be both beneficial and, in some cases, treatment-limiting. A sound knowledge of the underlying mechanism of action of the selected agent is required in order to understand treatment-associated changes in white blood cell counts, as well as monitoring consequences. This review is a comprehensive summary of the currently available DMTs with regard to their effects on lymphocyte count. In the first part, we describe important general information about the role of lymphocytes in the course of MS and the essentials of lymphopenic states. In the second part, we introduce the different DMTs according to their underlying mechanism of action, summarizing recommendations for lymphocyte monitoring and definitions of lymphocyte thresholds for different therapeutic regimens.

## 1. Introduction

As more treatment options emerge that have a significant impact on the peripheral immune system, the evaluation of lymphocyte count, and that of specific lymphocyte subsets, become more important in the treatment selection and management of patients with multiple sclerosis (MS) [1,2]. A greater understanding of the underlying pathophysiological mechanisms of MS has led to the development of therapeutics that address the cell count, migration, or functional state of lymphocytes. Though helpful in combatting the disease, changes in lymphocyte physiology can also be treatment-limiting. In addition, the measurement of peripheral lymphocyte counts appears to be important for treatment sequencing and planning of wash-out periods [3]. Pharmacological effects on lymphocytes in the peripheral blood can serve as markers of patient compliance and can also assist in understanding the mechanism of action of MS therapies [4,5].

Peripheral blood lymphocytes are frequently monitored in clinical practice as blood is easily accessible [6]: lymphocytes continuously enter and exit the lymphoid and non-lymphoid organs via the blood [7]. The assessment of lymphocyte subsets in the blood may provide useful information on immune system status [8]. The measurement of physiological parameters of lymphocyte subsets has been used for some time to assist the selection of treatment regimens in specific diseases, e.g., human immunodeficiency virus (HIV) infection [9]. However, blood lymphocytes can also be influenced by many conditions other than a disease or its treatment, including stress, smoking, sports, and aging [8]. The extent of variation caused by these different factors can easily obscure alterations that have diagnostic value in pathogenic conditions.

This review is an overview of the different treatment approaches in MS with respect to their effect on absolute and relative lymphocyte counts and their subsets. To assess the relevance and practical implications, we discuss the underlying mechanism of action and recommendations for treating lymphopenia.

## 2. General Information

### 2.1. Physiology of Peripheral Blood Lymphocytes

**Figure 1:**
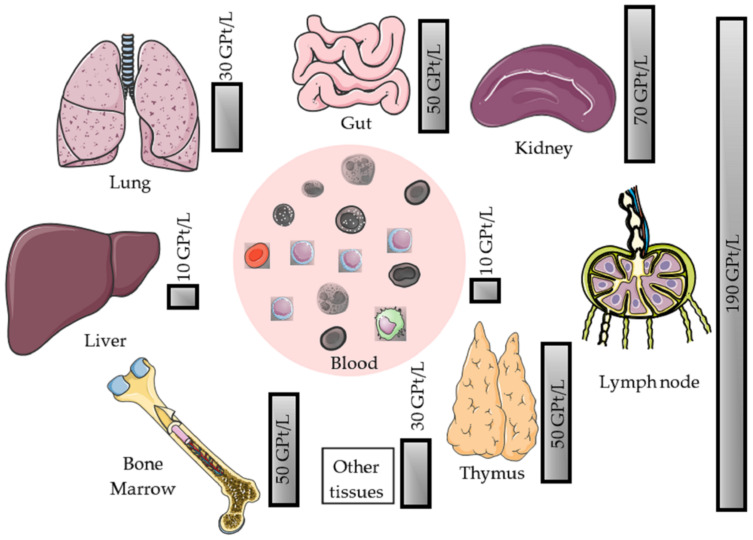
Differentiated depiction of the quantitative distribution of leukocytes in the human body. The various organ systems and lymphocytic compartments known to comprise relevant sources of leucocytes. The size of the associated boxes represents the quantity of stored leukocytes, which is also given in GPt/L.

Lymphocytes reside in different organs of the human body. They circulate through the primary lymphoid organs (thymus and bone marrow), the secondary lymphoid organs (spleen, lymph nodes (LN), tonsils, and Peyer’s patches (PP)), as well as non-lymphoid organs such as the blood, lungs, and liver. The distribution of leuko- and lymphocytes in the various organ compartments other than the central nervous system (CNS) should be considered when interpreting blood counts (Figure 1).

Lymphocytes circulating in the peripheral blood represent only about 2% of the total number of lymphocytes in the body of young adults. In blood, T lymphocytes make up most (60–80%) of the total peripheral lymphocyte count, with the rest comprising B lymphocytes and natural killer (NK) cells [10]. In physiological circumstances where the proliferation of lymphocytes in the blood is very low, their number depends on their exit from and entry into the blood, together with their transit through different organs. This situation is complicated by the fact that lymphocytes, like granulocytes, have a marginal pool that is in dynamic exchange with the peripheral blood lymphocytes [11]. Very rapid alterations in the number and composition of lymphocytes in the blood, e.g., from stress, are probably due to exchanges between the marginal pool lymphocytes and the peripheral blood lymphocytes [12]. Little is known about the size and location of the marginal pool and even less about the regulation of exchange. 

### 2.2. Role of Lymphocytes in the Pathogenesis of MS

For a deeper understanding of why MS therapeutics often focus on lymphocytes, directly or indirectly, one has to consider the lymphocyte-driven pathogenesis of the disease. In MS, the immunological compartment of interest is the central nervous system (CNS) beyond the blood-brain barrier. It is important to determine the factors involved in lymphocyte dynamics and their distribution between different immunological compartments before extrapolating data from peripheral blood analysis to the context of other organ systems in the body [13,14]. The cerebrospinal fluid (CSF) is a body fluid, which is both easily accessible and the most proximate to the pathological alterations of MS. Consequently, analysis of CSF provides an important window into the pathological underpinnings of MS [15,16]. In clinical practice, repeated CSF analysis is not feasible; therefore, despite the acknowledged limitations, peripheral blood lymphocytes are analyzed as a proxy.

### 2.3. Effects of Disease-Modifying Therapy (DMT) on Lymphocyte Number and Function

**Figure 2:**
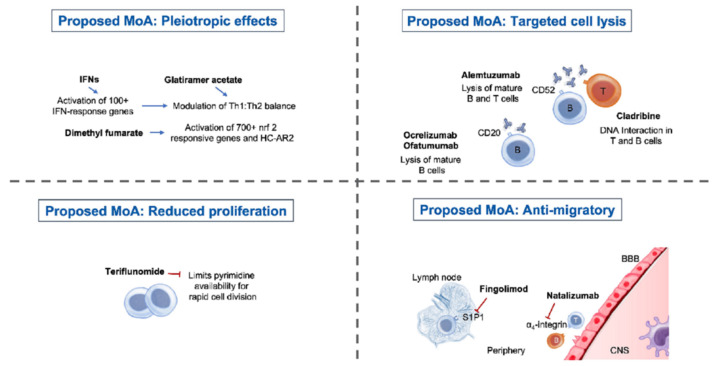
Major mechanisms of action (MoA) of different multiple sclerosis (MS) therapeutics. Pleiotropic effects are suggested by glatiramer acetate, interferon-beta-1a, and dimethyl fumarate. Teriflunomide interacts via blocking the dihydro-orotat-dehydrogenase lymphocyte proliferation. Induction treatment regimes that induce lysis of selected immune cells include the monoclonal antibody treatments alemtuzumab, ocrelizumab or ofatumumab, or antimetabolite cladribine. Inhibition of lymphocyte migration is seen in the sphingosine-1-phosphate (S1P)1-receptor modulators and the monoclonal antibody natalizumab [17,18]. BBB, blood-brain barrier; CNS, central nervous system; IFN, interferon; Th1/2, T helper 1/2 cells; nrf2, nuclear factor erythroid-derived 2-like 2.

Today, different MS treatment regimens are available that affect and modulate immune response by various mechanisms (Figure 2). Most of these treatments focus on lymphocytes, and potential side effects include lymphopenia, with rapid lymphocyte recovery after treatment cessation. When switching between treatments, a transition period should be considered depending on the treatment’s underlying mechanism of action and the recovery of individual lymphocyte counts. 

### 2.4. Definition of Lymphocytopenia (Lymphopenia)

Lymphocytopenia or lymphopenia is defined by abnormally low levels of lymphocytes in the blood [6]. Lymphopenia may be present as part of pancytopenia, in which the total numbers of all types of blood cells are reduced. In some cases, lymphopenia can be further classified according to which type of lymphocytes (T cells, B cells, NK cells) are depleted. 

**Figure 3:**
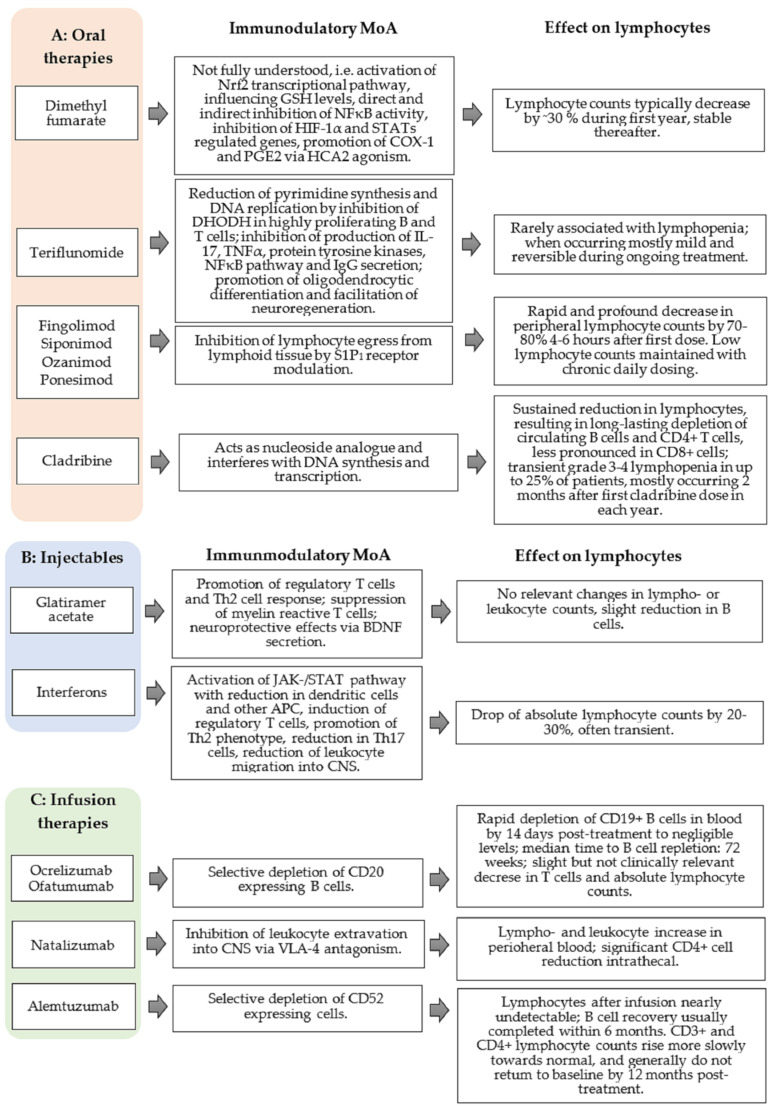
Association of proposed mechanism of action (MoA) of disease-modifying therapies (DMTs) and effects on lymphocytes. Categorized: oral therapies (**A**), injectables (**B**), and infusion therapies (**C**). CNS, central nervous system; COX-1, Cyclooxygenase-1; GSH, DHODH, dihydroorotate dehydrogenase; Glutathione; HCA2, hydroxy-carboxylic acid receptor 2, HIF-1α, hypoxia-inducible factor -1α; IL, interleukin; JAK/STAT, Janus kinases/signal transducer and activator of transcription proteins; nrf2, nuclear factor erythroid-derived 2-like 2; TNF-α, tumor necrosis factor-α; PGE2, prostaglandin E2; S1P, sphingosine-1-phosphate; Th1/2/17, T helper 1/2/17 cells; VLA, very late antigen.

Various treatments for MS have an impact on lymphocyte count and can account for relative and absolute lymphopenia, respectively (Figure 3) [19,20,21]. In addition, infections and other autoimmune diseases can also cause lymphopenia [6]. 

The normal laboratory range of lymphocytes is usually described using the 2.5th and 97.5th percentile, on the assumption that 2.5% of the population has abnormally low counts and 2.5% have abnormally high counts [22]. In a very large study conducted on the Danish population, the normal range for lymphocyte counts was defined as 1.1–3.7 GPt/L. To simplify and standardize classification, the World Health Organisation (WHO) has defined the lower limit of normal as 1.0 GPt/L or 1.0/mm^3^. The National Cancer Institute’s Common Terminology Criteria for Adverse Events (NCI-CTAE) is a frequently applied scoring system for grading the degree and severity of lympho- and leukocytopenia (Table 1).

In the general population, infection risk increases linearly below an absolute lymphocyte count of approximately 1.7 GPt/L. Even at a mild lymphocyte decrease (grade 1), there is a 26% higher risk of infection, at grade 2, there is a 44% increase in risk, and with grade 3, it increases rapidly (+76%) [23]. In addition, MS patients may already have lymphopenia prior to immunomodulatory treatment. In one study, lymphopenia was detected in 10% of treatment-naïve MS patients, which was no different from values in a matched cohort of healthy controls [23]. Further analysis revealed no association between pre-treatment lymphocyte count and patient variables, including age, sex, MS category, autoimmune comorbidities, disease duration, time since the last relapse, and last relapse severity. Importantly, pretreatment lymphopenia predicts post-treatment lymphopenia. Therefore, before starting immunomodulatory treatment for MS, it is important to identify at-risk patients that may need frequent lymphocyte monitoring. 

### 2.5. Potential Relevant Lymphopenia-Associated Complications

#### 2.5.1. Opportunistic Infections

Infections may run a more rapid and severe course in immunosuppressed patients than in those with normal immune function. Depending on the mechanism of immunomodulation, the risk of selected opportunistic pathogens should be considered [24,25]. Opportunistic infections are defined as infections with facultative pathogens such as distinct viruses, fungus, or protozoa that take advantage of a weakened immune system. Taking the prevailing pathogen spectrum into account, rapid induction of anti-infective measures or preventive application is required. Whereas neutrophil granulocytes play a key role in the defense of bacterial infections, lymphocyte function is especially important for the control of viral diseases. The risk of infection or reactivation of viral pathogens is notably increased in patients with lymphopenia. Lymphopenia is a particular risk factor for John Cunningham virus (JCV) reactivation and the development of progressive multifocal leukoencephalopathy (PML); however, the risk of opportunistic infections is generally lower with lymphopenia compared with other cytopenias (e.g., neutropenia). When starting an immunodepletive therapy, latent virus or mycobacterial infections should be ruled out, and vaccination should be considered.

#### 2.5.2. Immune Reconstitution Inflammatory Syndrome

Immune reconstitution inflammatory syndrome (IRIS) is a clinical deterioration in patients with opportunistic infections due to recovery of the immune system. The syndrome is well known in HIV patients during combined antiretroviral therapy. In certain circumstances, IRIS can become a severe complication in MS patients following treatment with DMTs. IRIS most commonly occurs following natalizumab-associated PML after cessation of therapy. The only known effective therapy for PML is reconstituting the immune system. The causal therapy must be discontinued, and elimination may be accelerated by plasmapheresis or immunoadsorption [26,27]. However, the rapid remission of lymphocytes and their ability for CNS transition prompts the evolution of IRIS. The histopathological hallmark of IRIS is an inflammatory lesion with a dense T cell infiltrate dominated by CD8+ T cells and numerous macrophages [28,29]. Within lesions, the JCV may be detected but may also be clear [29,30]. Ongoing PML infection and IRIS cannot be assessed by clinical examination, and even an MRI scan usually cannot differentiate between PML-associated IRIS and ongoing PML. In individual cases, a lumbar puncture or CNS biopsy should be considered to clarify the diagnosis.

#### 2.5.3. Secondary Autoimmunity

Secondary autoimmune syndromes are a further phenomenon associated with immune reconstitution after therapeutic lymphocyte depletion. In the setting of MS, these autoimmune phenomena may occur after lymphocyte depletion by the monoclonal antibody alemtuzumab or after bone marrow transplantation, which is a current strategy for treatment-refractory disease [31,32]. In up to one-third of patients, thyroid autoimmunopathy is the most common secondary autoimmune syndrome occurring during the phase of naive T cell expansion after lymphocyte depletion [33]. Other less common autoimmune syndromes include thrombocytopenic purpura and glomerular nephritis. The mechanisms responsible for reconstitution-associated autoimmune diseases are unclear but may include a relative bias towards a Th2-mediated immune response and reduced competition for autoreactive lymphocytes to expand during the time when recovery from lymphopenia begins [33].

### 2.6. Recommended Monitoring of Lymphocyte Count

Monitoring during DMT use in MS is intrinsically tied to the frequent evaluation of lymphocyte and lymphocyte subset counts. Based on the underlying mechanism of action, lymphocyte counts are affected differently, and the variation may be associated with treatment effects versus relevant side effects. Depending on the selected treatment strategy and proposed immunomodulatory effects, different lymphocyte thresholds are tolerated, and different monitoring regimes are recommended (Table 2). It is important to address these aspects as each DMT comes with its own individual monitoring plan (Table 3). The correct interpretation of lymphocyte count during immunomodulatory treatment for MS is important to enable individual clinical decision-making in everyday clinical practice. 

## 3. Disease-Modifying Drugs and Their Effects on Lymphocyte Count

### 3.1. Mechanism of Action: Immunomodulation

#### 3.1.1. Glatiramer Acetate

##### General Facts and Clinical Trial Data

Glatiramer acetate (GA) was the first DMT for MS successfully evaluated in humans (1977) and was approved by the US Food and Drug Administration in December 1996 and by the European Medicines Agency in 2001 for daily (20 mg/day) or triweekly (40 mg) subcutaneous application in patients with MS. Initially developed as a chemical and immunological analog of the major myelin antigen (myelin basic protein, MBP) to induce experimental autoimmune encephalopathy (EAE), GA did not work as intended. Instead of promoting encephalitic changes, GA was revealed as an efficient suppressor of encephalitic modulation. This effect and could even prevent EAE, which should normally be induced by myelin antigens such as GA [34]. Across five randomized controlled clinical trials, GA 20 mg has consistently demonstrated efficacy in reducing the annualized relapse rate (ARR 29%) and MRI disease activity (33% reduction in the total number of enhancing lesions) and slowing of disability progression in patients with relapsing-remitting MS (RRMS) [35,36]. Due to its favorable and well-characterized safety profile, GA is still often prescribed in patients with mild or moderate forms of MS.

##### Mechanism of Action and Impact on Lymphocyte Count

GA cross-reacts with MBP in a humoral and cellular respect and serves as an altered peptide ligand that promotes regulatory T cells instead of stimulating autoimmune T cell reactivity [34,37]. The immunological effect underlies a strong and effective binding of MHCII molecules on antigen-presenting cells (APC). They compete with MBP and other myelin proteins for binding sites [38,39]. This binding effectively replaces MBP, proteolipid protein (PLP), and MOG-derived peptides on their MHCII binding sites. This results in an altered T cell response, leading to suppression of myelin reactive T cells [39,40] and the emergence of regulatory Th2 cells, which are able to recognize GA as well as MBP to cross the blood-brain barrier and secrete anti-inflammatory cytokines [41,42]. These GA-specific Th2 cells additionally secrete high amounts of brain-derived neurotrophic factor (BDNF), which promotes neuroprotective effects [43]. Furthermore, GA functionally inactivates T cells by antagonism on the T cell receptor and can induce regulatory CD4+, CD25+ cells by activating the regulatory pathway protein FOXP3 (Figure 3B) [44]. 

While the total number of T cells in the blood compartment remains stable, studies have shown that GA treatment is associated with a reduction of B cells, plasma blasts, memory B cells, and a shift from pro- to anti-inflammatory B cell phenotypes [45]. This may be driven by the cross-reactivity of B cell receptors for GA with antigens that are expressed in MS lesions [45]. In contrast with interferon beta, GA is only associated with leukopenia or leukocytosis in exceptional cases [46].

##### Recommended Monitoring

Considering these rare cases of lymphopenia/leukopenia but also leukocytosis and thrombocytopenia, a regular check of blood counts should be done at least tri-monthly in the course of the first year of therapy (Table 2 and Table 3). Subsequently, laboratory intervals can be increased to once or twice per year in the case of normal blood counts. The risk of severe GA-associated infections is low and not clinically meaningfully increased (1–2%) [47]. 

Although there are no convincing study results regarding immune responses following vaccinations, GA treatment is not considered to limit immune responses [1]. Verifying sufficient vaccination response via titer recording should be considered. Patients receiving GA should not be vaccinated with attenuated vaccines.

#### 3.1.2. Interferons

##### General Facts and Clinical Trial Data

Interferons are a family of cytokines and physiologically function as signaling proteins. Since 1993 (US) and 1995 (EU), respectively, interferon-type beta (IFN-β) has played a role in the disease-modifying treatment of MS. Within the scope of the PRISMS study, subcutaneous (three times a week) application of INF-β-1a showed a risk reduction for relapses of 27% (22 µg, three times a week) and 33% (44 µg, three times a week) in a dose-related manner. Furthermore, it proved an effective treatment for RRMS in terms of defined disability and all MRI outcome measures [48]. Today there are various preparations that differ by mode and frequency of administration. In addition to RRMS, interferon is approved for the treatment of clinically isolated syndrome (CIS) and immunomodulation during pregnancy and breastfeeding [49,50].

##### Mechanism of Action and Impact on Lymphocyte Count

The effects of interferons are complex and, even today, are not completely understood. Activation of the JAK-/STAT-pathway via binding of the IFNAR-2 receptor is an established mechanism of action that leads to the expression of various genes (e.g., MX protein, beta2-microglobulin, 2′/5′-oligoadenylate synthetase, neopterin) [51]. The activation of the signal transduction by INF-β results in an antiviral, immunomodulatory, and antiproliferative effect [52].

With respect to the immunomodulatory impact, the following underlying mechanisms are considered (Figure 3B):

(a) IFN-β leads to a reduction of dendritic cells and down-regulates the antigen presentation by APCs in peripheral blood and in the CNS by microglia and monocytes.
(b) The expression of toll-like receptor (TLR) 3, TLR7, and myeloid differentiation primary response 88 (MyD88) on dendritic cells increases, which leads to an altered immune response.
(c) INF-β induces CD4+, CD8+, CD25+, FOXP3+, and FOXA1+ T cells (regulatory T cells). A reduced inflammatory T cell response is observed by inhibiting the stimulation and activation of T cells (e.g., by modulation of co-stimulating molecules on dendritic cells), inhibition of the expression of MHCII molecules, and co-stimulating factors like CD80 and CD28 on APC [53,54].
(d) The secretion of cytokines and chemokines is altered during IFN-β treatment (interleukin (IL)-10 and IL-4 increased; IL-2 and TNFα decreased). The differentiation of CD4+ cells shift from Th1 to a Th2 phenotype; thereby, resulting in a less pro-inflammatory but more anti-inflammatory cytokine milieu [55].
(e) The number of Th17 cells also decreases, leading to a reduction of IL-17 release and induction of apoptosis of autoreactive T cells [56,57].
(f) The effects on cytokines, chemokines, matrix metalloproteinase, and adhesion molecules (especially very late antigen [VLA]-4 on T cells) result in a reduced leukocyte migration via the blood-brain barrier into the CNS [53,58,59].

IFN-β-1a treatment results in selective, time-dependent effects on many cell populations in peripheral blood [60]. The IFN-β-promotes down-regulation of pro-inflammatory CD4+, CD8+ memory T cells, and memory B cells accompanied by an increase in regulatory T cells [52,53,58,61]. 

The majority of patients treated with IFN-β exhibit a fall in absolute lymphocyte counts of approximately 20–30% compared to the baseline value. About 15% of interferon-treated patients develop lymphocyte decreases below the lower limit of normal, 3.5% below 0.8 GPt/L, and about 1% of patients below 0.5 GPt/L [62]. The drop in lymphocyte count is often transient and recovers to normal levels within months. During a study evaluating the dynamics of lymphopenia during IFN-β treatment, onset of cytopenia occurred within the first 6 months of therapy in at least two-thirds of patients [62]. The majority of events were mild and generally resolved within 3–4 months while continuing therapy. Dose reductions were uncommon, and only a small proportion of patients (6 of 727; 0.8%) discontinued treatment after approximately 2 years because of hematological abnormalities when receiving the highest dose of INF-β-1a (44 μg three times weekly).

##### Recommended Monitoring

The rate of severe infections during IFN-β treatment does not seem to be significantly increased [1]. On the contrary, IFN-β has clear antiviral effects. There are no data available with respect to the duration of lymphocyte recovery in the case of lymphopenia. However, if repopulation has not occurred long after treatment discontinuation, hematological diseases should be excluded.

A regular check of blood counts including, leukocyte and lymphocyte counts, should be done at least tri-monthly in the course of the first year of therapy (Table 2 and Table 3). Subsequently, laboratory intervals can be increased to once or twice a year in the case of normal blood count levels. 

Although data regarding immune responses following vaccinations are lacking, it is not thought that humoral or cellular immune response to vaccination during IFN-β treatment might be impaired [63,64]. Verifying sufficient vaccination response via titer recording should be considered. Vaccinating patients during IFN-β treatment with attenuated live vaccines (e.g., varicella-zoster virus [VZV]) should be carefully considered.

#### 3.1.3. Dimethyl Fumarate

##### General Facts and Clinical Trial Data

Dimethyl fumarate (DMF) has been used to treat psoriasis since 1994. In 2013, it was finally licensed as an oral first-line treatment for MS, after two phase III clinical trials, DEFINE and CONFIRM, demonstrated clinical efficacy of DMF in RRMS by reducing the ARR and the mean number of new or enlarging MRI lesions throughout the course of the study [65,66,67]. DMF-treated patients receive two single daily doses of 240 mg. Common side effects are gastrointestinal complaints and intermittent flushing. 

##### Mechanism of Action and Impact on Lymphocyte Count

Although the precise mechanism of action is not completely characterized, there are currently at least five main mechanisms for the general action of DMF and its active metabolite, monomethylfumarate (MMF), that have been described so far (Figure 3A). These include:

(a) The activation of the nuclear factor erythroid-derived 2-like 2 (Nrf2) transcriptional pathway, which mediates the regulation of cellular antioxidant responses and stimulation of cytoprotective and anti-inflammatory factors such as heme oxygenase-1 (HO-1) [68,69];
(b) The regulation of cellular responses to oxidative stress via binding of DMF/MMF to thiol groups of glutathione (GSH) and therefore influencing intracellular GSH levels [70,71];
(c) The direct and indirect inhibition of NF-κB activity by DMF leading to altered cytokine production by APC, to the inhibition of Th1/Th17 responses and promotion of Th2 responses [72,73];
(d) The modulation of oxidative stress-sensitive transcription factors, hypoxia-inducible factor-1α (HIF-1α), and STATs by DMF mediating the inhibition of their regulated genes [70,74,75];
(e) Agonism of the hydroxy-carboxylic acid receptor 2 (HCA2) by MMF promoting the formation of cyclooxygenase-1 (COX-1) and prostaglandin E2 (PGE2) and the inhibition of neutrophil recruitment [76,77].

In both the DEFINE and CONFIRM studies, lymphocyte counts in DMF-treated patients declined by approximately 30% during the first year of treatment and remained stable thereafter [78]. Grade 3 lymphopenia, with <0.5 GPt/L, was seen in about 6% of the patients receiving DMF [67,79]. 

Unlike other DMTs in MS, a DMF-driven fall in lymphocyte count does not appear rapidly; however, it is often present within the first six months of DMF intake [46]. Despite the short pharmacological half-life of DMF, after therapy cessation, full lymphocyte regeneration takes several weeks or months in the majority of patients. The precise mechanism of fumaric acid-promoted lymphopenia is still unknown, but until now, apoptotic processes and depletion of lymphocytes have been assumed [80]. Among others, in vitro studies have shown that DMF induces T cell apoptosis with a preferential effect on memory T cells. Furthermore, DMF induces concentration-dependent apoptosis of B cells from healthy controls, with B cells of MS patients appearing to be more vulnerable [80]. During early DMF treatment, the dynamics of lymphocyte subsets change in the following way: B cell counts initially experience the greatest rate and proportion of decline, detected as early as four weeks after treatment initiation. By week eight, reduced circulating numbers of CD4+ and CD8+ T and NK cells can be observed. Consistent with the pattern in B cells, the decline in NK cell counts appears to stabilize after 12 weeks, remaining below normal, whereas CD4+ and CD8+ T cells counts continue to decline from baseline to week 24, whereby CD8+ T cells have the greatest median percentage reduction [81]. Overall, a significant reduction in the absolute counts of functional subsets can be observed at week 24, with the greatest median percentage reduction from baseline in T and B cell memory populations and the least effect on naive T and B cell subsets [81]. As studies show, early absolute lymphocyte count drop is associated with later development of severe, prolonged lymphopenia (<0.5 GPt/L for >6 months) while on treatment [66,67,82]. Known risk factors of this phenomenon are older age (>55 years), lower baseline absolute lymphocyte count, and recent natalizumab treatment [65].

##### Recommended Monitoring

After starting DMF treatment, a complete blood count should be performed every 6–8 weeks, as lymphocyte decline can be expected during the first weeks (Table 3). Less frequent monitoring of blood count is then needed in a 3–6 month interval, as lymphocyte counts normally remain stable after month 12. In the case of leukopenia of <3.0 GPt/L or lymphopenia of <0.5 GPt/L, DMF therapy should be discontinued (Table 2). In the case of grade 2 lymphopenia (0.5–0.8 GPt/L), continuous monitoring of blood counts and high vigilance for opportunistic infections, especially the development of PML, are required [1]. 

Although approval studies showed no increased risk of infection during DMF therapy in general, real-world data presented several cases of PML when taking fumaric acid derivates. Up to 11/2015 four PML cases, after long-duration DMF treatment, appeared without previous immunosuppression or other crucial immuno-compromising factors. As all of these patients were 50–70 years old, there seems to be an age-dependent effect. Furthermore, DMT-associated lymphopenia appears to develop predominantly in elderly patients. The role of lymphopenia in DMF-associated PML is not yet fully understood. While three of the DMF-treated patients with PML had lymphopenia < 0.5 GPt/L intermediately, one DMF-associated PML case only developed enduring grade 2 lymphopenia of about 0.6 GPt/L [83]. Although the causal relationship between DMF and the development of PML is not completely understood, continuous lymphopenia is a well-known risk factor for PML in general [83]. Nonetheless, it appears that PML can occur during DMF therapy even with moderate lymphopenia [84], requiring careful and frequent monitoring of blood counts. 

Limitations of vaccination in DMF-treated patients have not yet been evaluated [1]. However, the risk of attenuated live vaccines during DMF treatment should be thoughtfully weighed. To this date, there are no data for increased malignancy risk during DMF long-term therapy.

### 3.2. Mechanism of Action: Target Lymphocyte Proliferation

#### Teriflunomide

##### General Facts and Clinical Trial Data

Teriflunomide is an active metabolite of the prodrug leflunomide, which has been used in the treatment of rheumatoid arthritis as a DMT since 1988. In 2012, it was approved for the treatment of RRMS in the US (7 and 14 mg daily) and in 2013, in Europe (14 mg daily) [85] after efficacy and safety of teriflunomide were confirmed in the phase II trial, TEMSO and phase III clinical trials, TOWER and TENERE. Treatment significantly reduced ARR in MS patients by about 34% compared with placebo [86,87]. The simple application scheme of a once-daily oral intake led to a broad acceptance and compliance in patients, although typical adverse events like hair thinning, arthralgia, paresthesia, and persistent gastrointestinal complaints including nausea, diarrhea, or elevation of transaminases could be limiting. 

##### Mechanism of Action and Impact on Lymphocyte Count

Teriflunomide reversibly inhibits the dihydroorotate dehydrogenase (DHODH)—a mitochondrial enzyme involved in de novo pyrimidine synthesis and DNA replication of highly proliferating T and B lymphocytes (Figure 3A). By reducing pyrimidine de novo synthesis, the proliferation of activated B and T cells declines without prompting cell death. As resting T lymphocytes use nucleotides from degraded DNA and RNA, their survival does not depend on an intact DHODH function. The immunological protection against pathogens is thus ensured, whereas the damaging proliferation of activated autoimmunity driving B and T cells is reduced. In this way, a shift to regulatory T cell subtypes and a reduction in clonal diversity in the CD4+ T cell repertoire can be observed [88]. As teriflunomide crosses the blood-brain barrier [88], it might also be able to reduce microglia proliferation and induce the production of anti-inflammatory interleukins like IL-10 by microglia, which has been shown in vitro studies [89]. 

Besides the anti-proliferating effect, both leflunomide and teriflunomide inhibit the production of IL-17, TNFα, protein tyrosine kinases, the NFκB-pathway, and the immunoglobulin G (IgG) secretion of activated B cells [85,90,91]. Furthermore, teriflunomide induces apoptosis of Epstein-Barr virus (EBV)-transformed B cells [92] and seems to reduce glutamate levels and endotoxicity in the CNS [93]. Another favorable effect is the promotion of oligodendrocytic differentiation, the amelioration of axonopathy by attenuating CD8+ T cell cytotoxicity and supporting the proliferation of regulatory CD8+ T cells in the CNS [94,95], which facilitates neuroregeneration. 

The effect on circulating lymphocytes appears within the first six weeks of treatment [96]. The selective and reversible inhibition of mitochondrial DHODH results from targeted inhibition of proliferating lymphocytes in a decline of CD4+ and CD8+ T cells, memory B cells, and NK cells [97]. However, teriflunomide is rarely associated with lymphopenia and neutropenia. When it occurs, it is mostly mild and reversible during ongoing therapy or after discontinuation. In the patient populations of the TEMSO, TOWER, and TENERE studies, there was an overall decline in absolute lymphocyte counts from week 0 (1.89 GPt/L) to week 24 (1.67 GPt/L), remaining stable thereafter [98]. Mean counts generally remained above the lower limits of normal; however, grade 1 and 2 lymphopenia occurred in 7.3% and 2.2% of patients, respectively. No cases of grade 3 or 4 lymphopenia were reported in the pooled core studies [98]. The median treatment duration with teriflunomide prior to the development of lymphopenia was 17.9 weeks for grade 1 and 20.4 weeks for grade 2. The prevalence of lymphopenia during teriflunomide declined over time (up to 10.7 years follow-up); most events occurred in the first year of treatment. The median time to recovery from grade 1 lymphopenia during teriflunomide treatment was 10.6–11.1 weeks, and for patients with grade 2 lymphopenia, 16.6–49.9 weeks [98]. 2.3% of patients exposed to teriflunomide had grade 1 lymphopenia lasting longer than six months. The duration of grade 2 lymphopenia, however, did not persist for longer than six months. 

##### Recommended Monitoring

A complete blood count should be done every second month in the course of the first six months after starting teriflunomide therapy (Table 3). Subsequently, laboratory intervals can be increased to every three months in the case of normal lymphocyte and leukocyte counts. In the event of a lymphocyte decrease to <0.5 GPt/L, teriflunomide therapy should be discontinued (Table 2). In the rare case of critical lymphopenia and/or opportunistic infections during teriflunomide therapy, an accelerated elimination should be achieved with the oral administration of a bile acid sequestrant (e.g., 8 g cholestyramine three times daily for 11 days) as teriflunomide serum levels are detectable up to two years after discontinuation due to enterohepatic recirculation. The rate of severe infections with teriflunomide therapy is 1.4% (7 mg) and 2.2% (14 mg), respectively, versus 2.1% for placebo [96]. Patients treated with teriflunomide were able to mount sufficient immune response to vaccines, which, however, tended to be weaker than those in placebo-treated patients [99]. Immunization via live attenuated vaccines should be avoided.

### 3.3. Mechanism of Action: Target Lymphocyte Migration

#### 3.3.1. Sphingosine-1-Phosphate Receptor Modulation

##### General Facts and Clinical Trial Data

Fingolimod was the first sphingosine-1-phosphate (S1P)-receptor modulating agent approved in the USA in 2010 for relapsing MS after two phase III trials (FREEDOMS and TRANSFORMS) demonstrated potent efficacy, safety, and tolerability. Whereas fingolimod, as an unselective S1P receptor antagonist binds to four of the five known S1P receptors (S1PR_1–5_) and therefore exhibits a higher risk for adverse events (bradyarrhythmia, atrioventricular blocking, macular edema), second generation agents siponimod and ozanimod demonstrate favorable selectivity towards S1P_1_ receptors. A further S1PR_1_-selective agent, ponesimod, was recently approved in 2021. Siponimod is the first potential oral therapy for secondary progressive (SP) MS as the phase III trial EXPAND demonstrated a significant reduction in disability progression in SPMS patients compared with placebo [100]. Ozanimod and ponesimod both broaden the therapy range for active relapsing MS. 

##### Mechanism of Action and Impact on Lymphocyte Counts

Fingolimod, siponimod, ozanimod, and ponesimod are structural analogs of natural sphingosine phosphate [101]. In a phosphorylated state, fingolimod binds to four of the five known S1P receptors (S1PR_1_ and S1PR_3–5_) [102,103]. Siponimod, ozanimod, and ponesimod exhibit selective affinity for type 1 and 5 of the S1P receptors, leading to a lower risk of adverse events, such as bradycardia and vasoconstriction, mainly induced by S1PR_3_ activation. 

Binding with high affinity to S1PR_1_ expressed on lymphocytes, lymphocyte egress from lymphoid tissues into the peripheral compartment is inhibited by all approved S1P receptor modulators, preventing the infiltration of auto-aggressive lymphocytes into the CNS (Figure 3A) [104,105,106]. Initial receptor activation is, paradoxically, followed by S1PR_1_ functional antagonism. Accordingly, receptors are internalized and degraded, thus rendering lymphocytes unresponsive to the normal S1P gradient, which represents the obligatory signal that would ordinarily allow them to egress from lymphoid tissues [105,107,108]. Additionally, binding to S1P receptors expressed in the CNS (S1PR_1/5_) promotes a modulating effect on neurogenesis, neural function, and migration [109,110]. Fingolimod binds to S1PR_1/3_ on smooth muscle and endothelial cells, which influences vascular homeostasis and vascular permeability. Furthermore, fingolimod induces a negative chronotropic effect via S1PR on atrial myocytes [111,112].

As S1PR modulators inhibit CCR7+ lymphocyte egress from secondary lymphoid organs, resulting in a profound decrease in naive and central memory T cells and memory B cells in the periphery [113,114]. Treatment with fingolimod significantly decreases the absolute numbers of all major lymphocyte subsets, except for NK cells. The reduction is most pronounced within T helper and B cell populations [115]. Dramatic reductions within the naïve and central memory T cell populations can be found [115]; the reduction is less pronounced among effector memory cells. The number of regulatory T cells (Tregs) also decreases, but to a lesser extent than other T cell populations, resulting in a relative preservation of Tregs with a memory phenotype [115]. In summary, within T cells, naïve and central memory cells are most profoundly affected by a fingolimod-induced reduction, whereas memory Tregs are relatively preserved. 

A dose-dependent decrease in total peripheral lymphocytes by 70–80% can be observed, and most fingolimod-treated patients reach grade 2–4 lymphopenia after starting therapy. Grade 4 lymphopenia is a common adverse event occurring in 15–20% of patients [116,117]. In a German and Swedish cohort of fingolimod-treated patients with a low baseline lymphocyte count, women with a low body mass index were suggested to have a higher risk of lymphopenia [116]. A history of treatment with any IFN-β was significantly more frequent in patients who experienced lymphopenia [118]. This is because the IFN-β family influences the production of cytokines by lymphocytes and are considered to be related to myelosuppressive activities [119]. A study by Ohtani et al. showed that a low lymphocyte count at baseline and a treatment history of any IFN-β therapy is associated with grade 4 lymphopenia during fingolimod treatment [118]. Lymphocytes and their subsets return to the normal range around 1–2 months after treatment discontinuation [120]. Different studies discuss efficacy depending on T and B cell decreases during fingolimod therapy. Current real-world data show a wide range of peripheral lymphocyte counts during treatment, depending on the individual distribution of CD4+ and CD8+ T cells, CD19+ B cells, and NK cells. While peripheral CD4+ T cells and CD19+ B cells are markedly reduced by S1PR_1_ therapy, CD8+ T cells and NK cells are less affected and less relevant to variations in lymphocyte counts in individual patients [21]. 

It is assumed that withdrawal of fingolimod results in overexpression of lymphocytic S1PR_1_ leading to lymphocyte egress from lymph nodes and promoting disease rebound after treatment discontinuation [121]. Autopsy results from a patient who died after severe rebound relapse revealed increased S1PR_1_ immunoreactivity on hypertrophic astrocytes in tumefactive plaques, indicating that the withdrawal of fingolimod results in astrocytic overexpression of S1PR_1_ [122,123]. Due to the increased risk of more intense lymphopenia during fingolimod therapy, different treatment regimen alternatives have been assessed. However, the change from conventional therapy to intermittent dosing carries a risk of rebound, and the efficacy of an alternate-day fingolimod administration was not effective enough to inhibit disease activity [124,125]. 

Siponimod leads to a dose-dependent reduction of peripheral lymphocytes to 20–30% of baseline (median nadir approximately 0.56 GPt/L), with a recovery to the normal range within 10 days in 90% of patients after treatment discontinuation [126]. However, in some patients, lymphocyte recovery can take up to 3–4 weeks. In the pivotal phase III EXPAND study, grade 4 lymphopenia was observed in 1% of patients [126]. 

There are insufficient real-world data of lymphocyte count during ozanimod treatment. Combining data from the RADIANCE and SUNBEAM trials enabled a comparison of ozanimod to fingolimod, and analysis showed a higher absolute mean lymphocyte count (difference in means 0.4 GPt/L) during ozanimod treatment compared with fingolimod treatment [127]. During the RADIANCE study, ozanimod treatment led to dose-dependent suppression of absolute lymphocyte counts to <0.2 GPt/L in four participants (3.3%). These reductions were transient and not associated with infections or treatment discontinuation [128]. Early clinical studies of ponesimod therapy show an overall reduction of absolute lymphocyte count, compared to baseline, of about −1.3 GPt/L. Ponesimod treatment led to a marked reduction in overall T and B cell counts. Specifically, the number of CD4+ cells showed a significant drop, whereas CD8+ and NK cells were less affected [129]. Similar to siponimod and ozanimod, reliable real-world data for ponesimod are not yet available due to recent regulatory approval. 

Taken together, data on studies of siponimod, ozanimod, and ponesimod show a lower risk of higher-grade lymphopenia than for fingolimod, and this might be considered when selecting treatment alternatives where the desire is for fewer side effects. 

##### Recommended Monitoring

Before starting treatment with S1PR-modulators, chronic active infections should be excluded. Specifically, VZV status should be defined, and the evaluation of hepatitis B, C, and HIV should be considered. In the absence of VZV antibodies, patients should be immunized with VZV vaccine prior to treatment, which can be started four weeks after vaccination at the earliest.

Four weeks after the commencement of S1PR-modulators, a complete blood count should be performed (Table 3). Subsequent laboratory intervals can be increased to 3–6 months in the case of normal lymphocyte and leukocyte counts. In the case of peripheral lymphopenia < 0.2 GPt/L (confirmed by a second test after two weeks), S1PR therapy should be discontinued until lymphocyte counts reach levels > 0.6 GPt/L (Table 2).

In the case of acute infection, diagnostic and therapeutic measures should be adopted immediately, especially concerning viral herpetic infections (e.g., VZV infection or reactivation, Herpes simplex virus (HSV)-encephalitis), mycotic (e.g., cryptococcal meningitis), or bacterial infections (e.g., atypical mycobacteria). A higher risk of infections can be assumed considering the underlying mechanism of action. However, trial results suggest that for S1PR modulators, there is no direct correlation between absolute peripheral lymphocyte count and the likelihood of infective complications [117].

The risk of PML during S1P receptor modulator therapy is lower than that for natalizumab [130]. In most of the known cases, a ‘carry over’ mechanism following prior natalizumab therapy is assumed. There was no correlation to peripheral lymphopenia [130]. Frequent MRIs should be performed to assess the potential risk of PML, in addition to standard MRI MS monitoring. Regular evaluation of JCV-serostatus should be considered.

Efficacy of vaccination can be limited during, and up to two months after, therapy discontinuation. Immunization with live, attenuated vaccines should be avoided during S1PR modulator therapy. 

#### 3.3.2. Natalizumab

##### General Facts and Clinical Trial Data

Natalizumab was the first monoclonal antibody approved for the treatment of RRMS in 2004. The efficacy of natalizumab (300 mg i.v. every four weeks) has been demonstrated in two phase III trials (AFFIRM, SENTINEL) [131]. Despite the significant reduction in both relapse rate and the number of new T2 or gadolinium-enhancing MRI lesions [131,132], it did not achieve inhibition of disability progression [133]. After temporary withdrawal because of an accumulation of PML cases, natalizumab is available for the treatment of highly active RRMS, with consideration of PML predisposing risk factors. 

##### Mechanism of Action and Impact on Lymphocyte Counts

Natalizumab is a humanized recombinant IgG antibody that impairs leukocyte extravasation into the CNS and intestinal tract by blocking the alpha-4 subunit of the integrin molecules on leukocytes [134]. By inhibiting the interaction with the endothelial vascular cell adhesion molecule (VCAM)1 lymphocytes are not able to cross the blood-brain barrier, and inflammation in the CNS compartment is reduced (Figure 3C) [135]. As natalizumab is an IgG4 antibody, the binding does not result in lysis or destruction of the target cells, e.g., by complement factors [136].

In the brain tissue compartment, however, natalizumab leads to a significant reduction in CD4+ cells in cerebrospinal fluid, resulting in a reduction of the CD4+/CD8+ ratio [137,138] that is detectable up to 6 months after treatment cessation. In addition to inhibiting the migration of CD4+ cells into the CNS, natalizumab has other anti-inflammatory effects. These include a significant decrease of APC and dendritic cells in the perivascular space as well as the down-regulation of surface expression markers MHCII, which might also contribute to the long-lasting effect on CD4+ cell counts in the CNS [139]. Unlike other known DMTs, the administration of natalizumab leads to an increase in CD4+, CD8+ T cells, CD19+ B cells, and NK cells in serum without relevant effects on the CD4+/CD8+ ratio in peripheral blood, but with a reduction of this ratio in the CNS [140,141]. The increase of absolute lymphocyte counts in serum results from an increased release of CD34+ promotor cells from the bone marrow, on the one hand, and the impaired lymphocytes egress from the vessel into the brain tissue, on the other. The natalizumab-induced increase of peripheral lymphocytes stabilizes 3–6 months after starting treatment [140] and lasts up to 6 months after discontinuation [142].

##### Recommended Monitoring

During natalizumab therapy, a complete blood count should be performed every 3–6 months (Table 2 and Table 3). The peripheral increase of absolute leukocyte and lymphocyte count serves as a robust biomarker, indicating a sufficient VLA-4 antagonism [143,144]. In the case of clinical or subclinical disease activity, a lack of increase in lymphocyte cell count may indicate the appearance of neutralizing antibodies against natalizumab.

The altered immune-cellular milieu observed in the CNS up to 6 months after stopping natalizumab should be considered when changing therapy regimen, especially when switching to an immunodepletion therapy mechanism. However, severe rebound disease activity is a known phenomenon, especially longer than 3 months after natalizumab discontinuation. Taking into consideration the well-known dynamics of CNS cell changes after interruption of natalizumab therapy, the interval preceding immunodepletion therapy should be as long as possible but as short as necessary. Frequent MRI to assist individual decision-making for lumbar puncture can help detect the early return of disease progression versus PML in this wash-out period. In this context, vanishing lymphocytosis prior to the end of natalizumab therapy should raise awareness of neutralizing antibodies as a possible cause of sudden disease progression.

Although two studies did not show significant differences in the vaccine-specific antibody responses to several types of immunization, three other studies revealed restricted immune response following influenza vaccination in natalizumab-treated patients compared to healthy controls [145,146]. Immunization via live, attenuated vaccines should be avoided during natalizumab treatment.

### 3.4. Lysis of Specific Lymphocytes Subsets

#### 3.4.1. B Cell Depletion

##### General Facts and Clinical Trial Data 

We have learned that many of the underlying inflammatory processes in MS pathology appear to be B cell-mediated, evidenced by the development of oligoclonal bands in the CSF, the role of antigen presentation, antibody production, pathogenic cytokine release, and the formation of meningeal ectopic lymphoid tissues. These findings suggest that B cell depletion could be an effective treatment strategy for MS [147]. Ocrelizumab is a half-yearly intravenous humanized anti-CD20 monoclonal IgG1 antibody, which is approved for the treatment of active relapsing MS or primary progressive MS (PPMS). In clinical trials, ocrelizumab significantly reduced ARR relative to IFN-β-1a in RRMS patients and decreased the risk of disability progression relative to placebo in patients with PPMS (OPERA I + II, ORATORIO) [148,149]. Since the beginning of 2021, subcutaneous monthly ofatumumab—a humanized anti-CD20 monoclonal IgG1 antibody—has complemented the range of B cell-depleting therapies for MS.

##### Mechanism of Action and Impact on Lymphocyte Count

Ocrelizumab binds to the surface CD20-molecules and selectively depletes CD20-expressing B cells through antibody-dependent cell-mediated cytotoxicity, antibody-dependent cellular phagocytosis, complement-dependent cytotoxicity, and apoptosis (Figure 3C) [150,151]. The resulting decrease in the number and function of B cells promotes the chief immunomodulatory effect of ocrelizumab. However, additional, poorly understood mechanisms may also contribute to its clinical benefits [150]. As CD20 is expressed on pre-, mature, and memory B cells, but not on lymphoid stem cells, pre-existing humoral immunity due to plasma cells is preserved during ocrelizumab therapy [150]. Furthermore, innate [150] and adaptive [152] immunity remain unaffected after B cell depletion.

Ocrelizumab administered every 24 weeks decreases CD19 positive peripheral cells to negligible levels within 2 weeks, and this is sustained over 96 weeks of treatment [148,153,154]. In a phase II study [155] in patients with RRMS receiving four cycles of 600 mg ocrelizumab every 24 weeks, the median time to B cell repletion was 72 weeks after last administration [154]. CD19+ repopulation begins slowly at about 6 months after the last infusion. Depletion and repopulation of B cell subset data are not publicly available despite being part of the protocol of the developmental phase II extension trial [156]. With regard to the anti-CD20 antibody rituximab, repopulation of memory B cells would take significantly longer than CD19+ B cell repletion, which is largely driven by the repopulation of immature/mature B cells [156].

Very little is known about B cells in the central and secondary lymphoid immune compartments, unlike the peripheral blood compartment (Figure 2). A recent study revealed that a fraction of CD20+ B cells in the spleen are resistant to intravenous anti CD20 treatment [157]. After cessation of treatment, this population expanded in parallel to de novo B cell generation from bone marrow, resulting in an increased frequency of potentially pathogenic B cells containing a B cell-stimulating immunization. In this context, subcutaneous administration of anti CD20 antibodies, e.g., by ofatumumab, might target B cells most efficiently in draining lymph nodes and other lymphoid tissues, whereas the intravenous application of ocrelizumab exerts a more thorough effect on the removal of splenic B cells [158].

Although ocrelizumab selectively depletes CD20+ B cells and few CD20+ T cells, a decrease in the total lymphocyte count below the lower limit of normal can be observed in about 21% of ocrelizumab treated patients (most commonly grade 1 and 2 lymphopenia, 1% grade 3 lymphopenia (0.2–0.5 GPt/L), but not <0.2 GPt/L) [159]. Furthermore, peripheral T cell numbers can be modulated as well. It is thought that this effect is induced by an altered B cell cytokine and interleukin release as well as inhibited B cell/T cell interaction. Data from the ORATORIO clinical trial demonstrated that CD4+ T cells remained stable throughout the whole treatment period, whereas an initial decrease of 2–6% compared to baseline of peripheral blood counts, including CD3+ and CD8+ T cells, was seen at week two after the first infusion. CD8+ T cells showed an additional decrease of 6% until week 120 [160]. Even when not a focus of the treatment mechanism of action, peripheral T cell count is relevant for defining immune-competence in selected patients during B cell-depleting therapies.

##### Recommended Monitoring

During ocrelizumab therapy, a complete blood count should be done every 3 months and should include the status of peripheral T and B cell subtypes as well as immunoglobulin (Ig) levels (Table 3). Minimizing the risk of infectious complications, relevant humoral Ig deficiency (Ig < 3 g/L) and a significant decrease of CD4+ T cells (<0.250 GPt/L) should be ruled out during ocrelizumab and ofatumumab administration (Table 2). In case of a relevant and persistent CD4+ T cell decrease, antibiotic and antiviral prophylaxis should be considered to prevent opportunistic infections (e.g., herpes and pneumocystis jirovecii infection). In the case of acute infection, diagnostic and therapeutic measures should be adopted immediately, including postponing an upcoming regular infusion and delaying infusion interval, respectively.

Few patients develop neutralizing anti-drug antibodies—as pivotal trials have shown [159,161]. Despite the low incidence of these neutralizing antibodies, a sufficient B cell depletion can be documented as an efficiency control [161].

During ocrelizumab therapy, selected cases of PML after natalizumab pretreatment are known to underline the need for enhanced vigilance regarding this complication [130]. In clinical trials, the rate of malignant diseases was slightly higher in ocrelizumab-treated patients compared with the control group; therefore, preventive assessment for malignancy should be completed regularly.

Based on the mechanism of action, effective immune response following vaccination is limited due to B cell depletion [162]. Although recommended, vaccination status should be checked and administered if absent prior to therapy start. However, even if the immune response is decreased, vaccination during ocrelizumab therapy is recommended and should be completed 4–6 weeks before the next application [130]. Patients should not be administered live, attenuated vaccines during ocrelizumab or ofatumumab therapy.

#### 3.4.2. Alemtuzumab

##### General Facts and Clinical Trial Data

Camapth-1H, today known as alemtuzumab, is a depleting anti-CD52 monoclonal antibody that is used as a pulsed immune reconstitution therapy in MS. Alemtuzumab has been used as an experimental treatment for MS since 1991. Two randomized trials (CARE MS I and II) provided evidence on alemtuzumab’s efficacy, showing a reduction in ARR of about 49–55% and a reduction in disability progression over 6 months compared to IFN-β [163,164].

Currently, alemtuzumab is approved in many countries as an escalation therapy for adults with a highly active RRMS disease course. Patients typically receive two infusion courses of alemtuzumab 12 months apart (first year 5-day infusion course, 12 mg per day; second year, 3-day infusion course, 12 mg per day). In the case of recurring disease activity, additional 3-day retreatment cycles can be applied.

##### Mechanism of Action and Impact on Lymphocyte Count

Alemtuzumab targets the cell surface glycoprotein CD52, which is expressed by all T and B lymphocytes, monocytes, and eosinophils. Binding to CD52 leads to a depletion of the target cells by antibody-dependent cellular cytotoxicity and complement-dependent cytotoxicity. Alemtuzumab exerts its clinical efficacy by its specific pattern of depletion and repopulation of different immune cell subsets. Specific repopulation patterns seem to be responsible for the long-term efficacy, even years after the last alemtuzumab course (Figure 3C) [165,166].

Lymphocyte depletion is associated with the release of cytokines, including TNFα, IL-6, and IFN-γ, which peak 2–6 h after administration and are linked to the appearance of infusion-associated reactions [5,167]. Rapidly after alemtuzumab infusion, peripheral lymphocytes are nearly undetectable. Importantly, a few days after alemtuzumab infusion, T cell and B cell subsets, as well as NK cells and APC are decreased, which is critical for immunocompetence in the first weeks after alemtuzumab application. During one month follow-up, CD4 T cells, CD8+ T cells, and CD19+ B cells continued to reduce and fell to 5–15% of their baseline levels. This was in contrast to NK cell and APC levels, which stabilized after the initial treatment phase [168]. Alemtuzumab-associated depletion within secondary lymphoid tissue is likely to be less marked [168]. 

Lymphocyte reconstitution is guaranteed as CD52 is not expressed on hematological precursor lymphocytes. The degree of recovery varies by cell type: B cells recover rapidly, reaching baseline levels within 3–6 months and demonstrating over-repopulation of about 30% compared to baseline levels 6–12 months after the first alemtuzumab infusion [166]. However, the distribution of the B cell pool is altered far longer; mature naive cells (CD19+, CD23+, CD27-) dominate, whereas memory B cells (CD19+, CD27+) are depleted [169]. In contrast to CD19+ cells, B cell, CD4+, and CD8+ T cell lymphopenia are prolonged, taking up to 35 months to reach the lower limit of normal [20]. Twelve months after application CD4+ T cells are still reduced to 30.5% (0.275 GPt/L) compared to baseline; CD8+ cells to 58% (0.245 GPt/L) compared to baseline [166], dominated by a memory phenotype up to 12 months after alemtuzumab. 

##### Recommended Monitoring

For lymphopenia, evaluation of complete blood count should be done monthly in the course of at least 48 months after the last alemtuzumab dose (Table 2 and Table 3). Although mild to moderate infections are common after alemtuzumab [163,164,170], serious infections following treatment are rare [171]. Mild to moderate infections range from respiratory or urinary tract infections to herpetic infections. The risk of the latter is greatest in the first month post-treatment and can be reduced by the intake of antiviral prophylaxis [171]. There is an increased risk for infections with listeria monozytogenes that can occur prior to the first alemtuzumab infusion. Abstinence from raw meat, raw fish, and unpasteurized milk should be commenced 2 weeks prior to starting alemtuzumab and continued for 2–3 months after the last application. The appearance of neutralizing antibodies against alemtuzumab should be assessed in individual patients that receive alemtuzumab retreatment due to ongoing disease activity [172]. Monitoring of lymphocyte count and incomplete lymphocyte depletion should be considered in case of lacking efficacy.

A further important aspect following lymphopenia after alemtuzumab is the risk of developing other autoimmune diseases, including autoimmune thyroid dysfunction, idiopathic thrombocytopenic purpura (ITP), or glomerulonephritis. Autoimmunity arising in the setting of T cell lymphopenia is a well-recognized clinical phenomenon [173,174]. The mechanistic aspects of this are not yet fully understood, but it is known that homeostatic proliferation of T cells after induction of lymphopenia relies on stimulation through T cell receptor-self peptide components, resulting in an oligoclonal population of cells skewed towards self-reactivity [175,176,177]. Regular assessment of thyroid hormones, renal function parameters, and platelet count is necessary.

Vaccine efficacy can be limited during the first months after alemtuzumab therapy [130]. Vaccinations status should be checked and administered when absent 6 weeks before starting alemtuzumab. In particular, VZV-negative patients should be immunized against VZV before starting alemtuzumab therapy. Booster vaccinations are recommended 6 months after each alemtuzumab application at the earliest. If necessary, verifying sufficient vaccination response via titer recording should be considered [130].

#### 3.4.3. Cladribine

##### General Facts and Clinical Trial Data

In 1980, cladribine was approved by the FDA for the treatment of hairy cell leukemia, originally as a parenteral formulation. Since then, efficacy has also been reported in a number of other hematologic malignancies and autoimmune diseases. Safety and efficacy of parenteral cladribine in patients with RRMS have been shown in several clinical studies, including three randomized, double-blind, parallel-group, placebo-controlled phase II/III trials [178,179]. A newer, orally administered formulation of cladribine has been evaluated in the 96-week phase III, double-blind, placebo-controlled, multicenter CLARITY study [180]. In CLARITY, oral cladribine led to a relative reduction in the ARR (reduction of approximately 55–58%), the risk of 3-month confirmed disability progression (reduction of around 31–33%), and in MRI active lesions (reduction of up to 88%) [180]. The most common adverse event in the cladribine treated group was mild or moderate lymphopenia, which was anticipated and inherent to the mechanism of action of cladribine [180].

##### Mechanism of Action and Impact on Lymphocyte Count

Cladribine (or 2-chloro-2’deoxy-b-D-adenosine) is a synthetic deoxyadenosine analog. Substitution of a hydrogen atom with chlorine at the 2-position of the purine ring makes the nucleoside analog resistant to degradation by adenosine deaminase, thus enabling cladribine to enter the cell via nucleoside transporter proteins. Inside the cell, cladribine is activated through phosphorylation by the enzyme deoxycytidine kinase (DCK). The preferential effect on lymphocytes is explained by a high concentration of DCK and low concentration of de-phosphorylating enzymes compared to other cells that lead to an intracellular accumulation of activated cladribine [181]. The exact mechanism of action of cladribine remains unknown. Assumptions suggest that accumulating cladribine interferes with the repair of single-stranded DNA breaks, leading to cell death [182]. In proliferating cells, it can also be incorporated into the DNA, impairing transcription. Furthermore, cladribine causes apoptosis via the caspase system [183]. These cytotoxic mechanisms interfere with DNA synthesis, repair, and therefore target both proliferating and resting lymphocytes (Figure 3A) [184]. Cladribine can cross the blood-brain barrier, and additional control of disease activity is thought to be achieved by the reduction of CNS-resident immune cells. [184,185]. Like alemtuzumab, cladribine is a pulsed immune reconstitution therapy. The recommended cumulative dose is 3.5 mg/kg over 2 years, administered as one treatment course of 1.75 mg/kg per year. Each treatment course consists of two treatment weeks, one at the beginning of the first month and one at the beginning of the second month of the respective treatment year. Each treatment week consists of 4 or 5 days on which a patient receives 10 or 20 mg as a single daily dose, depending on body weight. Following completion of the two treatment courses, no further cladribine treatment is required in years 3 and 4. Re-initiation of therapy after year 4 has not been studied.

The intake of cladribine leads to a sustained reduction in lymphocytes, resulting in the long-lasting depletion of circulating CD4+ T cells [181,186]. Median CD4+ T cell populations in cladribine-treated patients reach a nadir at around 4 months and then gradually increase again [184]. After treatment in the second year, a nadir is reached at about 60 weeks, with full reconstitution no later than 4 years after the first dose [184]. The decrease in CD8+ T cells is less pronounced, reaching a nadir at 4 months in the first year, and does not fall below the threshold of 0.2 GPt/L [184,187].

**Figure 4:**
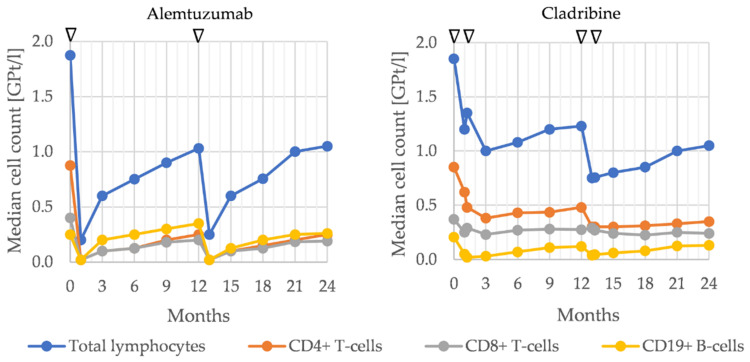
Schematic comparison of lymphocyte dynamics during treatment with alemtuzumab versus cladribine (adapted from [187] and [188]). The median total lymphocyte count (blue), CD4+ T-cells (orange), CD8+ T-cells (grey), and CD19+ B-cells (yellow) in GPt/L over a time period of 24 months after alemtuzumab application compared to cladribine therapy. Triangles indicate the time-point of treatment application.

Within the B cell population, magnitude and kinetics of depletion show a variation depending on the enzyme stocking of the B cell subpopulation. With the exception of plasma cells, mature, memory, and germinal center B cells show high levels of DCK and low potential of dephosphorylating enzymes, resulting in rapid and long-lasting depletion [184]. In summary, the dynamics of lymphocyte counts after cladribine intake show similarities with those during alemtuzumab therapy. Differences can be seen in a quicker reduction of T and B lymphocytes with alemtuzumab compared with cladribine and the overshoot of B lymphocyte repopulation with alemtuzumab, which is not seen after cladribine (Figure 4).

##### Recommended Monitoring

As cladribine’s mechanism of action is closely linked to a reduction in lymphocyte count, a regular complete blood count should be performed (Table 3). Lymphocyte counts should be defined before initiating cladribine in treatment years one and two, respectively, along with 2 and 6 months after the start of treatment in each treatment year [130]. If the lymphocyte count is below 0.8 GPt/L, the next cladribine pulse should not be started and active monitoring is required until values increase again (Table 2). In the case of not reaching a lymphocyte count of at least 0.8 GPt/L within 18 months after cladribine start, continuation is not recommended [130]. More careful monitoring of hematological parameters is recommended in the case of concomitant substances that affect the hematological profile (e.g., carbamazepine).

Patients with lymphocyte counts below 0.5 GPt/L should be actively monitored for signs and symptoms of infection, in particular, herpes zoster [130]. If symptoms occur, anti-infective treatment should be initiated as clinically indicated. In addition, monthly complete blood count and lymphocyte subsets are recommended in these cases. Weekly evaluation of complete blood count and lymphocyte subsets along with virostatic prophylaxis (e.g., acyclovir) is also suggested in patients with lymphocyte counts <0.2 GPt/L.

As cladribine-associated lymphopenia may increase the likelihood of infections, screening for latent tuberculosis, hepatitis B, C, and HIV, should be performed prior to initiation of therapy in years 1 and 2. In the clinical study database of cladribine in MS, PML has never been reported [130]. However, a baseline MRI scan should be performed before initiating cladribine, and yearly during follow-up.

Since cladribine induces a decrease in circulating B and T cells, limited immune response following vaccination can be assumed during and after treatment [130]. Therefore, recommended vaccinations should be administered prior to starting cladribine [1]. VZV-negative patients should be immunized before cladribine start. Booster vaccinations are recommended at the earliest, 6 months after each cladribine dose.

### 3.5. New Treatment Options under Investigation: Bruton’s Tyrosine Kinase Inhibitors—Non-Cell-Depleting Alternative to B Cell Modulation

Considering the role of B cells in MS pathogenesis and the therapeutic impact of anti-CD20+ monoclonal antibodies, other anti-B cell alternatives have been explored to prevent the problems associated with chronic B-cell depletion, such as humoral deficiency [189,190]. One promising non-cell-depleting alternative to B cell modulation is Bruton’s tyrosine kinase inhibitors (BTKi).

Bruton’s tyrosine kinase (BTK) is a key signaling node downstream of the B-cell receptor (BCR) and the receptors for the Fc region (FcR) of Igs, which mediate activation and a variety of effector functions in B lymphocytes and myeloid cells. In B cells, in particular, BTK is required for B-cell proliferation and differentiation into memory cells and antibody-producing cells [191]. Furthermore, an important function of BTK in B cells is its proposed requirement for BCR-mediated antigen presentation to T cells. BTKi presents an interesting opportunity to inhibit B cell pro-inflammatory functions without the risks associated with cell depletion. Regarding MS pathophysiology, the size of these so-called small-molecule agents is another advantage, as they are able to cross the blood-brain barrier.

Evobrutinib is a BTKi under investigation for MS. Detailed results are available from a recently published phase II placebo-controlled clinical trial of evobrutinib [192]. The drug is under further investigation in an ongoing phase III trial for relapsing MS patients. In a phase II clinical trial of patients with RMS, a further BTKi, tolebrutinib, met primary and secondary endpoints defined by the reduction of new gadolinium-enhancing lesions or enlarging T2 lesions. Tolebrutinib is currently under investigation in a phase III clinical trial for relapsing and progressive forms of MS. Fenebrutinib also completed a phase II clinical trial and is set to be evaluated in phase III trials for relapsing and progressive MS. Orelabrutinib—a potent, second-generation BTKi developed for B cell malignancies and autoimmune diseases, including MS, is undergoing a phase II randomized, double-blind clinical trial for patients with RRMS [190,193].

A 24-week placebo-controlled trial has provided data on lymphocyte variation during BTKi use. Here, the number of patients with a lymphocyte decrease was comparable in all groups (grade 1 lymphopenia). Selected patients presented grade 2 lymphopenia in the high-dose group of evobrutinib compared to lower doses or placebo [192].

At present, published data on experience with BTKi is primarily limited to experimental models of several human autoimmune diseases. Clinical experience in this field is very limited as BTKis are still in the early stages of development with many ongoing clinical trials. The first results are promising, but further, robust clinical research is needed.

## 4. Summary

Current DMTs in MS are often associated with changes in peripheral lymphocyte count. Regular, standardized monitoring of these peripheral lymphocyte and subset counts is essential to identify relevant side effects early. However, the underlying mechanism of action of a treatment regimen should be considered in order to understand its effect on lymphocyte count. Here we have demonstrated that different treatment regimens impact immune function and, specifically, lymphocyte count and their subsets in quite different ways. This insight has an important role in routine clinical practice for monitoring and interpreting peripheral white blood cell measures as the standard of care to determine treatment efficacy, patient compliance, treatment sequencing, and wash-out periods for treating MS patients.

## Figures and Tables

**Table 1:** Adapted classification of lymphocyte and leukocyte counts according to National Cancer Institute Common Terminology Criteria for Adverse Events (NCI-CTAE). LLN, lower level of normal.

Grade	NCI-CTAE Definitions of Severity for Adverse Reactions	Leukocyte Count	Lymphocyte Count	CD4 Lymphocyte Count
1	Mild, with no or mild symptoms; no interventions required	LLN–3.0 GPt/L	LLN–0.8 GPt/L	LLN–0.5 GPt/L
2	Moderate; minimal intervention indicated; some limitation of activities	<3.0–2.0 GPt/L	<0.8–0.5 GPt/L	<0.5–0.2 GPt/L
3	Severe but not life-threatening; hospitalization required; limitation of patient’s ability to care for him/herself	<2.0–1.0 GPt/L	<0.5–0.2 GPt/L	<0.2–0.05 GPt/L
4	Life-threatening; urgent intervention required	<1.0 GPt/L	<0.2 GPt/L	<0.05 GPt/L
5	Death related to adverse event			

**Table 2:** Recommended lymphocyte thresholds for disease-modifying therapies.

	Drug Name	Recommendations for Lymphocyte Cut-Off Values
Oral therapies	Dimethyl fumarate	Complete blood count every 6–8 weeks in first year of treatment, subsequently every 3–6 months, discontinuation of therapy in case of leukopenia of <3.0 GPt/L or lymphopenia of <0.5 GPt/L, in case of grade 2 lymphopenia (0.5–0.8 GPt/L) continuous control of blood counts and high vigilance for opportunistic infections
	Teriflunomide	Regular check of blood counts every second month in the first six months, subsequently every three months in the case of normal lymphocyte and leukocyte counts; therapy discontinuation in case of lymphocyte decrease < 0.5 GPt/L
	Fingolimod Siponimod Ozanimod Ponesimod	Regular check of blood counts 4 weeks after starting therapy, subsequently in case of normal lymphocyte and leukocyte counts, every 3–6 months; in case of repeated peripheral lymphopenia < 0.2 GPt/L, therapy discontinuation until lymphocyte counts reach levels > 0.6 GPt/L
	Cladribine	Regular complete blood count prior to cladribine intake and 2 and 6 months after start of treatment in each treatment year, in case of lymphocytopenia < 0.8 GPt/L, the next cladribine pulse must not be started and active monitoring is required until values increase again; in case of not reaching a lymphocyte count of at least 0.8 GPt/L within 18 months after cladribine start, continuation is not recommended
Injectables	Glatiramer acetate	Regular check of blood counts at least 3 monthly in first year of therapy, subsequently once or twice a year; in case of lymphopenia < 0.5 GPt/L discontinuation of therapy
	Interferons	Regular check of blood counts at least 3 monthly in first year of therapy, subsequently once or twice a year; in case of lymphopenia < 0.5 GPt/L discontinuation of therapy
Infusion therapies	Ocrelizumab Ofatumumab	Regular check of blood counts 3 monthly, including status of peripheral T and B cell subtypes as well as immunoglobulin levels, relevant humoral immunoglobulin deficiency (Ig < 3 g/L), and significant decrease of CD4+ T cells (<0.250 GPt/L) should be ruled out
	Natalizumab	Regular check of blood counts every 3–6 months, peripheral increase of absolute leukocyte and lymphocyte count can serve as a biomarker, indicating sufficient VLA-4 antagonism
	Alemtuzumab	Regular complete blood count monthly in the course of at least 48 months after last alemtuzumab application

Ig, immunoglobulin; VLA, very late antigen.

**Table 3:** Recommended monitoring of lymphocyte counts for disease-modifying therapies.

			Months of Treatment												
	Drug Name	Predose	1	2	3	4	5	6	7	8	9	10	11	12	Post Month 12
Oral therapies	Dimethyl fumarate	X		X		X		X		X		X		X	every 3–6 months
	Teriflunomide	X		X		X		X			X			X	every 3 months
	Fingolimod Siponimod Ozanimod Ponesimod	X ^a,b^	X		X			X			X			X	every 3–6 months
	Cladribine	X ^c^		X				X						X	before initiating treatment in year 2, 2 and 6 months after start of treatment cycle in each year ^d^
Injectables	Glatiramer acetate	X			X			X			X			X	once or twice a year
	Interferons	X			X			X			X			X	once or twice a year
Infusion Therapies	Ocrelizumab Ofatumumab	X			X			X			X			X	every 3 months
	Natalizumab	X ^a^			X			X			X			X	every 3–6 months
	Alemtuzumab	X	X	X	X	X	X	X	X	X	X	X	X	X	monthly for at least 48 months after last application

CBC: complete blood count; WBC: white blood cells. ^a^ Washout period following previous treatment must be sufficient for lymphocyte count recovery. ^b^ Recent CBC (within the last 6 months) or after prior therapy discontinuation before treatment initiation. ^c^ Lymphocyte counts must be normal before initiating cladribine in year 1 and ≥0.8 GPt/L before initiating cladribine in year 2. If recovery takes >6 months, do not administer further cladribine therapy. If lymphocytes < 0.2 GPt/L, consider anti-herpes prophylaxis during time of grade 4 lymphopenia. If lymphocytes < 0.5GPt/L/L, actively monitor for signs/symptoms suggestive of infection, particularly herpes zoster. If such signs and symptoms occur, initiate anti-infectives as clinically indicated. Consider interruption or delay of cladribine until proper resolution of infection. ^d^ If lymphocytes < 0.5 GPt/L, actively monitor until values increase again (treatment course in year 2 may be delayed for ≤6 months to allow for lymphocyte recovery. “X” marks the time of monitoring.

## Data Availability

Not applicable.

## Abbreviations

AIDS	Acquired Immune Deficiency Syndrome
ALC	Absolute lymphocyte count
APC	Antigen-presenting cells
ARR	Annualized relapse rate
BDNF	Brain-derived neurotrophic factor
BTKi	Bruton’s tyrosine kinase inhibitor
CCR7+	C-C chemokine receptor type 7
CI	Confidence interval
CIS	Clinically isolated syndrome
CNS	Central nervous system
COX-1	Cyclooxygenase-1
CSF	Cerebrospinal fluid
DCK	Deoxycytidine kinase
DHODH	Dihydroorotate dehydrogenase
DMF	Dimethyl fumarate
DMT	Disease-modifying therapy
DNA	Deoxyribonucleic acid
EAE	Experimental autoimmune encephalopathy
EBV	Epstein-Barr virus
EDSS	Expanded Disability Status Scale
e.g.,	Exempli gratia
EU	European Union
FDA	Food and Drug Administration
FOXA1+	Forkhead box protein A1
FOXP3	Forkhead box P3
GA	Glatiramer acetate
GSH	Glutathione
HCA2	Hydroxy-carboxylic acid receptor 2
HIF-1α	Hypoxia-inducible factor -1α
HIV	Human immunodeficiency virus
HO-1	Heme oxygenase-1
HR	Hazard Ratio
HSV	Herpes simplex virus
i.e.,	Id est
i.v.	Intravenous
IFN	Interferon
IFNAR	Interferon-alpha/beta receptor
Ig	Immunoglobulin
IgG	Immunoglobulin G
IL	Interleukin
IRIS	Immune reconstitution inflammatory Syndrome
ITP	Immune thrombocytopenic purpura
JAK/STAT	Janus kinases/signal transducer and activator of transcription proteins
JCV	John Cunningham virus
LLN	Lower limit of normal
LN	Lymph nodes
MBP	Myelin basic protein
MMF	Monomethylfumarate
MHC	Major Histocompatibility Complex
MMF	Monomethylfumarate
MOG	Myelin oligodendrocyte glycoprotein
MRI	Magnetic Resonance Imaging
MS	Multiple sclerosis
MX1	Interferon-induced GTP-binding protein Mx1
MyD88	Myeloid differentiation primary response 88
NCI-	National Cancer Institute Common
CTAE	Terminology Criteria for Adverse Events
NF-κB	Nuclear factor ‘kappa-light-chain- enhancer’ of activated B-cells
NK cells	Natural killer cells
Nrf2	nuclear factor erythroid-derived 2-like 2
PGE2	Prostaglandin E2
PLP	Proteolipidprotein
PML	Progressive multifocal leukoencephalopathy
PP	Peyers patches
PPMS	Primary progressive multiple sclerosis
RNA	Ribonucleic acid
RRMS	Relapsing remitting multiple sclerosis
S1P	Sphingosine-1-phosphate
SPMS	Secondary progressive multiple sclerosis
TCR	T-cell receptor
Th1/2 cells	T helper 1/2 cells
TLR	Toll-like receptor
TNF-α	Tumor necrosis factor-α
Tregs	Regulatory T cells
US	United States
VCAM	Vascular cell adhesion molecule
VLA	Very late antigen
vs.	Versus
VZV	Varicella-zoster virus
